# Recombinant Protein Expression System in *Corynebacterium glutamicum* and Its Application

**DOI:** 10.3389/fmicb.2018.02523

**Published:** 2018-10-26

**Authors:** Min Ju Lee, Pil Kim

**Affiliations:** Department of Biotechnology, The Catholirc University of Korea, Bucheon, South Korea

**Keywords:** *Corynebacterium glutamicium*, expression host systems, cytosolic expression, secretory expression, recombinant protein, surface displayed expression

## Abstract

*Corynebacterium glutamicum*, a soil-derived gram-positive actinobacterium, has been widely used for the production of biochemical molecules such as amino acids (i.e., L-glutamate and L-lysine), nucleic acids, alcohols, and organic acids. The metabolism of the bacterium has been engineered to increase the production of the target biochemical molecule, which requires a cytosolic enzyme expression. As recent demand for new proteinaceous biologics (such as antibodies, growth factors, and hormones) increase, *C. glutamicum* is attracting industrial interest as a recombinant protein expression host for therapeutic protein production due to the advantages such as low protease activity without endotoxin activity. In this review, we have summarized the recent studies on the heterologous expression of the recombinant protein in *C. glutamicum* for metabolic engineering, expansion of substrate availability, and recombinant protein secretion. We have also outlined the advances in genetic components such as promoters, surface anchoring systems, and secretory signal sequences in *C. glutamicum* for effective recombinant protein expression.

## Introduction

Recombinant proteins, including biologics and enzymes, are useful in the biopharmaceutical, food, and chemical industries (Butenas, 2013). To date, more than 400 recombinant biologics have been approved by the US Food and Drug Administration (FDA), and more recombinant biologics are in the clinical development stage (Sanchez-Garcia et al., 2016). The demand for new biologics (such as antibodies, growth factors, and hormones) for the treatment of severe chronic diseases (such as cancer, anemia, and multiple sclerosis) has increased, and the market for recombinant proteins is expected to grow over the next few decades (https://www.grandviewresearch.com/press-release/global-protein-expression-market). *Corynebacterium glutamicum*, which is a non-lethal and non-emulsifying gram-positive bacterium, exhibits a low protease activity in the culture supernatant and can secrete protease-sensitive proteins into the culture supernatant (Liu et al., 2013). Absence of lipopolysaccharide (endotoxin) in *C. glutamicum*, which is the gram-negative bacterial surface component that should be removed for the production of therapeutic proteins (Srivastava and Deb, 2005), may increase the heterologous protein yield by minimizing the purification steps. *C. glutamicum* has generally been used as a generally recognized as safe (GRAS) host for the industrial production of biochemicals including L-glutamate and L-lysine (Lee et al., 2016). As a result, *C. glutamicum* is favorable for producing high yields of proteins that are difficult to secrete in the host and the proteins that must remain active in a non-pathogenic environment.

The industrial production of biochemicals including nutraceuticals has been established using *C. glutamicum* as a host (Nakayama et al., 1961). Since *C. glutamicum* was first isolated as an L-glutamate producer by Kinoshita and Udaka in 1956 (Kyowa Hakko Bio Ltd. Co., Japan) (Kinoshita et al., 1957), many L-amino acids have been produced using this soil bacterium. In addition, many biochemicals (biopolymers, organic acids, rare sugars, etc.) have been commercially produced from metabolically engineered *C. glutamicum* strains. The metabolic processes of *C. glutamicum* may be rationally modified for the production of various biochemicals using three approaches: (1) amplification of biosynthetic pathway enzymes to increase target products, (2) reduction of by-product formation, and (3) introduction of important enzyme feedback controls to optimize target biomaterials. All these approaches involve the use of recombinant protein expression in the cytosol to produce beneficial biochemicals.

This review summarizes the recent studies on the heterologous expression of the recombinant protein in *C. glutamicum* for various applications including metabolic engineering, expansion of substrate availability, and recombinant protein secretion. It also lists the advancements of genetic components for effective recombinant protein expression.

## Cytosolic protein expression in *C. glutamicum* for metabolic engineering

A common method for producing biochemicals from *C. glutamicum* is the overexpression of enzymes involved in the biosynthetic pathway of the target product in cytosol (Table 1), which involves recombinant protein expression. Jensen and Wendisch overexpressed the ornithine cyclodeaminase (OCD) gene from *Pseudomonas putida* for the production of L-proline, which is a biochemical that is typically used as a commodity chemical or feed additive; this overexpression resulted in an increased product yield of 0.36 g proline/substrate (Jensen and Wendisch, 2013). Another foreign protein (D-lactate dehydrogenase) from *Lactobacillus delbrueckii* was expressed to address the limitations of using lactic acid bacteria, which require a relatively expensive complex medium for D-lactate production, and Okino et al. reported a high level of D-lactate production in *C. glutamicum* (Okino et al., 2008).

**Table 1:** Examples of cytosolic protein expressions in *Corynebacterium glutamicum* for productions of biochemicals.

**Recombinant Protein**	**Product**	**Applications**	**Source**	**Producer**	**Titer (g/L_medium_)**	**Productivity (g/L/h)**	**Yield (g_product_/g_substrate_)**	**References**
**A. L-AMINO ACIDS AND RELATED BIOCHEMICALS**								
Alanine dehydrogenase (AlaD)	L-Alanine	Supplement in animal feed	*Lysinibacillus sphaericus*	R Δ*ldhA Δppc Δalr* + AlaD + GapA	98	3.1	0.83	Jojima et al., 2010
Glyceraldehyde 3-phosphate dehydrogenase (GapA)			*Corynebacterium glutamicum*					
Ornithine acetyltransferase (ArgJ)	L-Citrulline	Intermediate in the arginine biosynthesis, health, and nutrition applications	*Corynebacterium glutamicum*	ATCC 13032 Δ*argG ΔargR* + ArgJ	8.5	0.1	0.11	Zhang et al., 2018
Hemoglobin (Vgb)	L-Glutamine	Flavor enhancer	*Vitreoscilla*	ATCC14067 + GlnA (Y405F) + Vgb	17.3	0.36	0.08	Liu et al., 2008
3-deoxy-D-arabino-heptulosonate 7-phosphate synthase (DS), Chorismate mutase (CM), Prephenate dehydratase (PD)	L-Phenylalanine	Aromatic amino acids	*Corynebacterium glutamicum*	KY10865 + DS + CM + PD	28	0.35	0.47	Ikeda and Katsumata, 1992
Oornithine cyclodeaminase (ArgB)	L-Proline	Pharmaceutical and osmotic applications and feed additive	*Pseudomonas putida*	ATCC13032 Δ*argR ΔargF* + ArgB (A49V, M54V)	12.7	0.52	0.36	Jensen and Wendisch, 2013
Transketolase (TK)	L-Tryptophan	Supplement in animal feed	*Corynebacterium glutamicum*	KY9218 + DS + PGD + TK	58	0.73	0.25	Ikeda and Katsumata, 1999
3-eoxy-D-arabino-heptulosonate 7-phosphate synthase (DS), Chorismate mutase (CM)	L-Tyrosine	–	*Corynebacterium glutamicum*	KY10865 + DS + CM	26	0.32	0.43	Ikeda and Katsumata, 1992
**B. ORGANIC ACIDS**								
D-lactate dehydrogenase (D-LDH)	D-Lactate	Food packaging	*Lactobacillus delbrueckii*	R Δ*ldhA* + D-LDH	120	4	0.8	Okino et al., 2008
Glyoxylate reductase (YcdW)	Glycolate	Cosmetic industry to improve skin texture and to treat skin diseases	*Escherichia coli*	ATCC13032 Δ*aceB icd*GTG + YcdW	5.3	0.1	0.18	Zahoor et al., 2014
*Cis*-aconitate decarboxylase (CAD1)	Itaconic acid	Synthesis of resins, lattices, fibers, detergents, cleaners, and bioactive compounds	*Aspergillus terreus*	ATCC13032 *icd* A1G + MalE + CAD1 (optimized)	7.8	0.27	0.03	Otten et al., 2015
Acetohydroxy acid synthase (IlvBN), Acetohydroxy acid isomeroreductase (IlvC), Dihydroxy acid dehydratase (IlvD)	2-Ketoisovalerate	Precursor of L-valine, L-leucine, and pantothenate synthesis; substitute for L-valine or L-leucine in chronic kidney disease patients	*Corynebacterium glutamicum*	ATCC13032 Δ*ltbR ΔilvE ΔprpC1 ΔprpC2 +* PgltA mut_L1 + IlvBN + IlvC + IlvD	35	0.79	0.15	Buchholz et al., 2013
Isopropylmalate synthase (leuA)	2-Ketoisocaprate	Therapeutic agent	*Corynebacterium glutamicum*	VB +IlvBN + IilvC + IlvD + leuA (G462D)	9.2	0.37	0.24	Bückle-Vallant et al., 2014
Alcohol dehydrogenase (ADH)	12-Ketooleic acid	Plasticizers, lubricants, detergents, cosmetics, and surfactants.	*Micrococcus luteus*	ATCC13032 + GFP + ADH	–	1.2	74%	Lee et al., 2015
**C. POLYMERS**								
Lysine decarboxylase (CadA)	Cadaverine	Replacement for the oil-derived hexamethylenediamine for polyamide 66 (nylon 66)	*Escherichia coli*	ATCC13032 Δ*hom* + AmyA + CadA	22.9 mM	–	–	Tateno et al., 2009
Glutamate decarboxylase (GadB)	Gamma-aminobutyric acid (GABA)	Foods and pharmaceutical products	*Escherichia coli*	WJ008 + GadB mutant (Glu89Gln/Δ452-466 gene)	9.4	–	–	Choi et al., 2015
β-ketothiolase (PhaA),	Poly-hydroxyalkanoate (PHA)	Alternative to plastics	*Ralstonia eutropha*	ATCC13869 + PhaA + PhaB +PhaC	6	–	–	Matsumoto et al., 2011
NADPH-dependent acetoacetyl-CoA reductase (PhaB), P(3HB) synthase (PhaC),								
L-ornithine decarboxylase (SpeC)	1,4-Diaminobutane (putrescine)	Precursor of L-arginine and L-ornithine biosynthesis	*Escherichia coli*	ATCC13032 Δ*argR ΔargF* + SpeC + 5′21-ArgF (synthetic 5′-region)	19	0.55	0.16	Schneider et al., 2012
**D. RARE SUGARS**								
Rhamnulose-1-phosphate aldolase (RhaD)	D-Sorbose	Food additives, cancer cell suppressors, and building blocks for anticancer, and antiviral drug	*Escherichia coli*	SY6 + RhaD + YqaB (*lac* promoter)	19.5	–	–	Yang et al., 2015
Fructose-1-phosphatase (YqaB)	D-Psicose	Food additives, cancer cell suppressors, and building blocks for anticancer, and antiviral drug	*Escherichia coli*	SY6 + RhaD + YqaB (lac promoter)	13.4	–	–	Yang et al., 2015
D-galactose isomerase (D-GaI)	D-Tagatose	Functional sweetener	*Geobacillus thermodenitrificans*	PICG (Permeabilized and immobilized) + D-GaI	165	55	0.55	Shin et al., 2016
GDP-D-mannose-4,6-dehydratase (Gmd), GDP-4-keto-6-deoxy-D-mannose-3,5-epimerase-4-reductase (ManB), Phosphomanno-mutase (WcaG), GTPmannose-1-phosphate guanylyl-transferase (ManC)	Guanosine 50-diphosphate (GDP)-L-fucose	Precursor of fucosyl-oligosaccharides	*Escherichia coli*	ATCC13032 Gmd + WcaG + ManB + ManC	0.086	0.001	–	Chin et al., 2013
**E. ALCOHOL**								
Pyruvate decarboxylase (Pdc), Alcohol dehydrogenase (AdhB)	Ethanol	Alternative transportation fuel	*Zymomonas mobilis*	R Δ*ldhA Δppc* + Pgi + PfkA + GapA + Pyk + Glk + Fba + Tpi + Pdc + AdhB	119	2.3	0.48	Jojima et al., 2015

Jojima et al. designed protein expression systems as a way to reduce by-product formation in L-alanine production (Jojima et al., 2015). In a *C. glutamicum* strain, genes involved in the organic acid biosynthetic pathway (Δ*ldhA*: lactate dehydrogenase; Δ*ppc*: phosphoenolpyruvate carboxylase; Δ*alr*: alanine racemase) were inactivated; however, the *alaD* of *Lysinibacillus sphaericus* (encoding L-alanine dehydrogenase) along with the *gapA* of *L. sphaericus* (encoding glyceraldehyde 3-phosphate dehydrogenase promoting glucose consumption) were overexpressed, leading to a metabolic flux from organic acids to L-alanine. As a result, a high product (L-alanine) concentration (98 g/L_medium_) was obtained.

As a large amount of oxygen and energy is required in the production of L-amino acids in *C. glutamicum* (Kwong and Rao, 1991), Liu et al. reported a novel approach for improving the intracellular oxygen supply by expressing hemoglobin (Liu et al., 2008). They modulated the metabolism to increase the productivity of L-glutamate by inducing metabolic flux into the tricarboxylic acid (TCA) cycle and additionally expressed the hemoglobin protein of *Vitreoscilla* sp. (VHb) in *C. glutamicum* to increase the oxygen and energy supply, resulting in the increased production of L-glutamine.

In addition, cytosolic protein expression in *C. glutamicum* has contributed to the production of biochemicals such as polyhydroxyalkanoate (PHA) (Matsumoto et al., 2011), ethanol (Jojima et al., 2015), and γ-aminobutyric acid (GABA) (Choi et al., 2015). The industrial techniques for the production of rare saccharides such as D-tagatose (Shin et al., 2016), D-sorbose and D-psicose (Yang et al., 2015), D-allose (https://patents.google.com/patent/WO2017111339A1/en), and GDP-L-fucose (Chin et al., 2013) are also the methods of nutraceutical production that involve cytosolic protein overexpression in *C. glutamicum*.

## Surface-displayed enzyme expression in *C. glutamicum*

### Present applications of surface-displayed systems

The cell surface display systems can be used for a wide range of biotechnological and industrial applications: (1) a live vaccine that induces antigen-specific antibody responses by exposing heterologous epitopes to pathogenic bacterial cells (Lee et al., 2000); (2) screening of displayed peptides by sequential binding and elution (Boder and Wittrup, 1997); (3) expression of surface antigens to produce polyclonal antibodies in animals (Martineau et al., 1991); (4) using biological adsorbents for the removal of heavy metals (Bae et al., 2000); (5) using biological adsorbents for the removal of herbicides and environmental pollutants (Dhillon et al., 1999); (6) detecting single amino acid changes in target peptides after random mutagenesis; and (7) using biosensors with immobilized enzymes, receptors, or other signal-sensitive components (Aoki et al., 2002).

Purified enzymes have been used in many industrial bioconversion processes as immobilized enzyme catalysts. The immobilization of enzymes is time-consuming and costly because it involves several steps: growth of culture, disruption of cells, purification of enzymes, and immobilization of enzymes. When an enzyme is expressed on the cell surface of a microorganism as a whole-cell catalyst, additional purification steps are unnecessary and the whole-cell catalyst can be used repeatedly.

### Development of surface-display systems

The first surface expression system was developed in the mid-1980s to attach small peptides to proteins fused to bacteriophage surfaces (Smith, 1985). Thereafter, various phage display systems have been developed to express heterologous proteins on the surface of phages; however, the size of exogenous proteins that can be expressed on the surface is limited (Li, 2000). For example, foreign proteins that can be expressed in M13 phages have a length of 6.6 nm (Lee et al., 2012), while most enzymes are more than 10 nm in diameter (http://book.bionumbers.org/how-big-is-the-average-protein).

**Figure 1:**
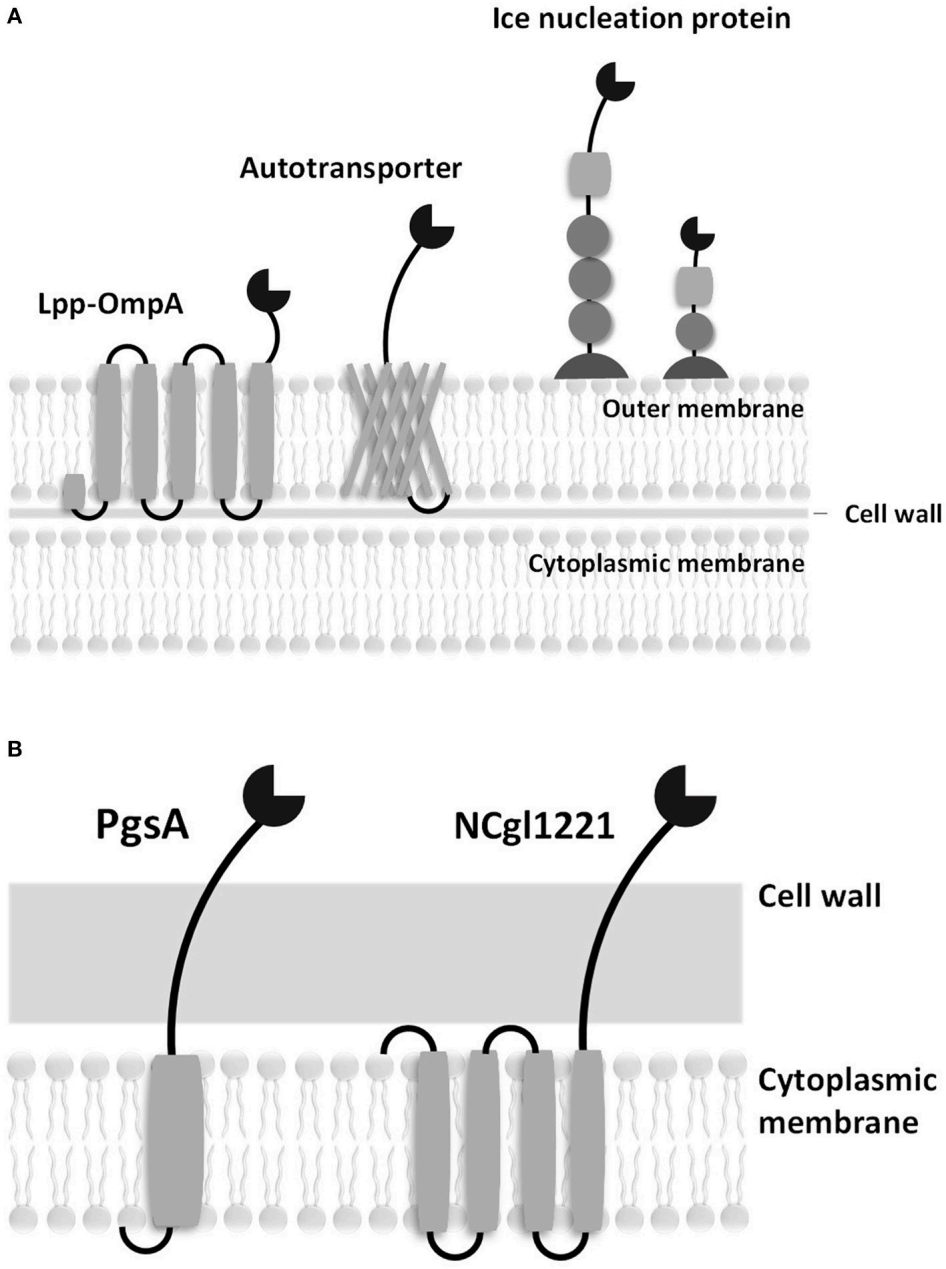
Schematic diagrams of surface displayed enzymes in bacteria. **(A)** Surface display systems of gram-negative bacteria. **(B)** Surface display systems of gram-positive bacteria.

To address this problem, microbial cell surface display systems have been developed (i.e., *C. glutamicum* is 2,000–6,000 nm in length and 500 nm in diameter). Microbial cell surface display is generally accomplished by expressing a passenger protein on the cell surface fused with preexisting microbial surface proteins or with anchoring motifs of the membrane protein (Figure 1). A C-terminal fusion, N-terminal fusion, or sandwich fusion strategy can be considered, depending on the characteristics of the fixed motif and the target protein. A good anchoring motif should meet the following requirements: (1) the premature fusion protein must have an efficient signal peptide or transport signal to pass through the inner membrane, (2) an anchoring motif must have a strong immobilization structure to retain the fusion protein on the cell surface, (3) an anchoring motif must be compatible with the inserted or fused foreign sequences (i.e., the anchoring motif should not become unstable following the insertion or fusion of heterologous sequences), and (4) an anchoring motif must be resistant to attack by proteases present in the periplasmic space or media. In cell display systems, the properties of the target protein are known to significantly affect transport to the cell surface. In particular, the folded structure of the target protein (e.g., a disulfide bridge) in the outer membrane (the periplasmic side) can affect the movement of the target protein (Maurer et al., 1997). In addition, the insertion of amino acid sequences that contain multiple charged residues or hydrophobic residues within the target protein can result in ineffective sequence secretion in bacterial hosts.

Good hosts for surface display should be compatible with the expressed protein and should be easily culturable without cell lysis. In addition, the host cells must have a low extracellular protease activity. Gram-negative bacteria, including *Escherichia coli*, have a complex surface structure, which consists of the cytoplasmic membrane, periplasm, outer membrane, and many anchoring motifs that have been developed based on the outer membrane proteins. Therefore, the anchoring motif fused with the target protein in gram-negative bacterial hosts must be transferred to the outer membrane through the cytoplasmic membrane and periplasm. On the other hand, gram-positive bacteria are considered to be more suitable for whole-cell catalysts and whole-cell adsorbents because of the robust structure of their cell walls. Many surface proteins can be covalently immobilized on the cell walls of *Bacillus* spp., *Staphylococcus* spp., and *C. glutamicum*. A eukaryotic GRAS host, *Saccharomyces cerevisiae*, has protein folding and secretion systems that are similar to those in mammalian cells; it has been reported that mammalian proteins could be linked to the cell wall via a glycosylphosphatidylinositol (GPI) anchor or disulfide bonds (Lee et al., 2003).

### Examples of surface-displayed system in *C. glutamicum*

The use of *C. glutamicum*, an important industrial biochemical producer and as a Gram-positive bacterial host, is advantageous in a cell surface display system because of the presence of various enzymes on the surface of *C. glutamicum* cells that extend the range of carbon sources during the production of biochemicals (Table 2). Starch is used as an industrial carbon source for microorganisms; however, *C. glutamicum* cannot consume starch directly. Starch should be provided in a hydrolyzed form using α-amylase or glucoamylase. Tateno et al. have described the use of starch as a carbon source directly by a surface-displayed enzyme on *C. glutamicum* (Tateno et al., 2007). PgsA, a transmembrane protein derived from *Bacillus subtilis*, is a part of the poly-γ-glutamate synthetase complex. It was used to anchor the α-amylase from *Streptococcus bovis* 148 on the cell surface. The resulting display system was able to produce L-lysine (yield of 6.04 g/L_medium_) from starch. In addition, a system displaying α-amylase fused with PgsA as an anchor produced 6.4% of poly-β-hydroxybutyrate (PHB) (Song et al., 2013) in a metabolically engineered *C. glutamicum*, using starch as raw material.

**Table 2:** Examples of surface-displayed enzyme expressions in *Corynebacterium glutamicum* for expansion of substrate availability.

**Passenger protein**	**Anchor protein**	**Substrate**	**Product**	**Resource**	**Producer**	**Titer (g/L_medium_)**	**Productivity (g/L/h)**	**Yield (g_product_/g_substrate_)**	**References**
**FOR AMINO ACIDS PRODUCTION**									
α-amylase (AmyA)	PgsA	Starch	L-Lysine	AmyA: *Streptococcus bovis* 148 PgsA: *Bacillus subtilis*	ATCC13032 Δ*hom +* PgsA + AmyA	6.04	0.25	0.18	Tateno et al., 2007
α-amylase (AmyA)	NCgl1221	Starch	L-glutamate	AmyA: *Streptococcus bovi*s 148 NCgl1221: *Corynebacterium glutamicum*	ATCC13869 + NCgl1221 + AmyA-FLAG	19.3	0.74	0.64	Yao et al., 2009
β-glucosidase (Sde1394)	Porin (*porC*)	Cellobiose	L-Lysine	β-glucosidase: *Saccharophagus degradans*	ATCC13032 Δ*hom +* PorC + Sde1394-FLAG	1.08	0.01	0.05	Adachi et al., 2013
β-glucosidase (Sde1394)	Porin (*porC*)	Cellobiose	L-Lysine	β-glucosidase: *Saccharophagus degradans*	DM 1729 + PorC + Sde1394	0.73	0.01	0.03	Anusree et al., 2016
α-amylase (AmyA)	Short-length (1–50) NCgl1337	Starch	L-Lysine	AmyA: *Streptococcus bovis* 148	ATCC13032 + NCgl1337 (Full length) + AmyA	10.8	0.6	0.29	Choi et al., 2018
**FOR POLYMERS PRODUCTION**									
α-amylase (AmyA)	PgsA	Starch	Polyhydroxybutyrate (PHB)	AmyA: *Streptococcus bovis* 148 PgsA: *Bacillus subtilis*	ATCC13032 Δ*hom +*PgsA + AmyA + PhaC + PhaA + PhaB	6.4wt%	0.88	1.6	Song et al., 2013
β-xylosidase (Xyl)	PorH	Xylooligosaccharides	1,5-diaminopentane (cadaverine)	*Bacillus subtilis*	PIS8 + PorH +Xyl + XylAB (*E. coli*) + ldcC	0.12	–	0.01	Imao et al., 2017
**FOR ORGANIC ACIDS PRODUCTION**									
α-amylase (AmyA)	PgsA	Starch	Lactate	AmyA: *Streptococcus bovis* 148 PgsA: *Bacillus subtilis*	ATCC13032 + PgsA + AmyA	6	0.6	0.65	Tsuge et al., 2013
			Succinate			1.5	0.15	0.16	
			Acetate			0.7	0.07	0.07	

In addition to PgsA, porin has been used as an anchor protein in *C. glutamicum*. Porin is a cell wall-related protein of *C. glutamicum* that is present in the mycolic acid layer. Adachi et al. have produced 1.08 g/L_medium_ of L-lysine from a cellobiose carbon source using a system in which β-glucosidase was displayed using PorC as an anchor protein (Adachi et al., 2013). In addition, Imao et al. have reported the display of β-xylosidase on the cell surface using PorH as an anchor protein (Imao et al., 2017). In this expression system, xylooligosaccharides were used as a carbon source to produce 0.12 g/L_medium_ of 1,5-diaminopentane (cadaverine).

The use of other anchor proteins has also been reported by Yao et al. They displayed *S. bovis* 148 α-amylase on the cell surfaces using the C-terminally truncated NCgl1221 anchor protein. In this system, 19.3 g/L_medium_ of L-glutamate was produced from starch (Yao et al., 2009).

Recent reports of Choi et al. suggested that the proteins from 19 known mycolic acid layers in the extracellular membrane of *C. glutamicum* can be used as anchoring motifs in surface display systems (Choi et al., 2018). The α-amylase of *S. bovis* was screened using a portion of NCgl1337 as an anchoring motif; this portion has a signal peptide and a predicted O-mycoloylation site. As a result, 10.8 g/L_medium_ of L-lysine was obtained from starch; this result demonstrates the potential of whole-cell biotransformation using the cell membrane proteins of *C. glutamicum*.

## Secretions of proteins from *C. glutamicum*

### Characteristics of natural secretion systems in *C. glutamicum*

The direct secretion of proteins into the culture medium by *C. glutamicum* over protein expression in the cytosol has several advantages. First, it is easy to obtain the target protein by purification because it does not require cell disruption and there are fewer proteins in the culture medium than in the cytoplasm (Nguyen et al., 2007). In addition, the oxidative environment of the extracellular culture fluid is suitable for the formation of disulfide bonds, which leads to protein folding and the expression of the active protein form (Makrides, 1996). Furthermore, the low extracellular protease activity of *C. glutamicum* contributes to the stability of the target proteins (Suzuki et al., 2009).

**Figure 2:**
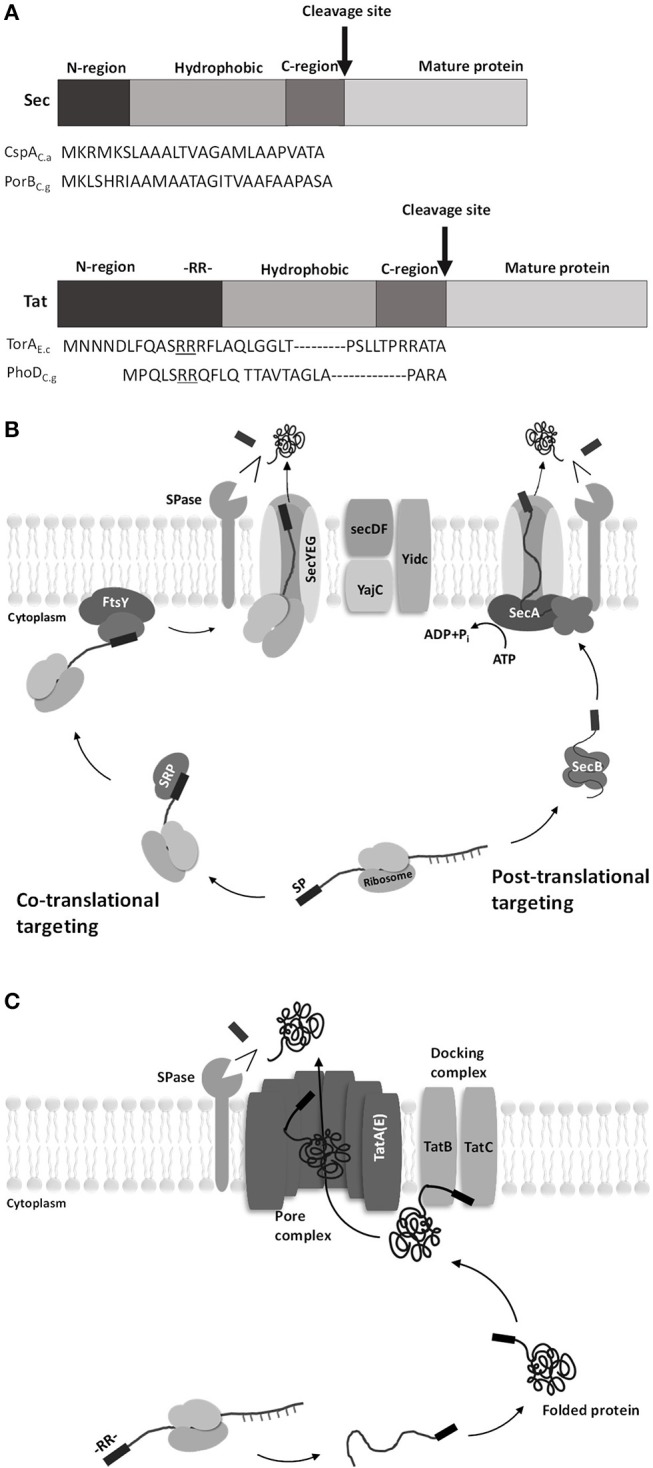
Diagrams of signal peptides for Sec pathway and Tat pathway in *C. glutamicum*. **(A)** General structure and amino acid sequence of the Sec- and Tat-type signal peptides. The signal peptide consists of three regions: the amino-terminal region (N-region), the hydrophobic region, and the carboxy-terminal region (C-region). The difference between the two pathways is that the N-region of the twin-arginine translocation (Tat)-type signal peptide is longer than the secretory (Sec)-type signal peptide because the Tat-type signal peptide contains a conserved twin-arginine residue (RR) at the end of the N-region [CspA_C.a_, surface (S)-layer protein from *Corynebacterium* *ammoniagenes*; PorB_C.g_, porinB from *C. glutamicum*; TorA_E.c_, TMAO reductase from *Escherichia coli*; PhoD_C.g_, alkaline phosphatase from *C. glutamicum*] (Berks et al., 2000). **(B)** Protein translocation by the Sec pathway. Sec translocase consists of the following components: SecYEG, a core protein in Sec translocase that forms the transmembrane protein-conducting channel (PCC), and SecDF, interacts with YajC to improve protein transport efficiency driven by the proton motive force (Scotti et al., 1999). In the co-translational targeting Sec pathway, signal recognition particles (SRPs) bind to the signal peptide at the beginning of translation where proteins are still bound to ribosomes. Then, the SRPs and the initial ribosomal protein (nascent protein) migrate to the SRP receptor and membrane protein FtsY and subsequently come in contact with SecYEG. The nascent protein passes through SecYEG while the ribosome is attached. In the post-translational targeting Sec pathway, a translation-finished protein binds to SecB without ribosome and then migrates to SecA-SecYEG complex. The delivered protein then passes through SecYEG while SecA is attached. **(C)** Protein translocation by the Tat pathway. The Tat system consists of TatA-like proteins (TatA, TatB, and TatE) and TatC (TatE seems to have the same function as TatA, though the difference is not clear yet). Translocation begins when the folded cargo proteins interact with the docking complex. The twin-arginine (RR) motif of the Tat signal peptide attaches to the signal peptide-binding loop of TatBC. The docking complex recognizes the cargo protein and inserts it into the membrane. TatA receives the cargo protein from the docking complex, and the cargo protein is translocated across the active pore complex. The signal peptide is then cleaved by type I signal peptidase, and the mature protein is separated from the cell membrane (Tuteja, 2005).

Two major translocation pathways have been known identified in *C. glutamicum*: the secretory (Sec)-pathway and the twin-arginine translocation (Tat)-pathway. The Sec-pathway transports unfolded proteins, whereas the Tat-pathway transports folded proteins (Kudva et al., 2013). These two pathways have the signal peptides necessary for the protein to pass through the cell membrane (von Heijne, 1985). The difference between the signal peptides of the two pathways is that the N-region of the Tat-type signal peptide is longer than that of the Sec-type signal peptide because the Tat-type signal peptide contains a conserved twin-arginine residue (RR) at the end of the N-region (Berks et al., 2000; Figure 2A).

The Sec-pathway is a system that secretes proteins in an unfolded state (Figure 2B). Sec-dependent protein secretion systems have a co-translational targeting system and a post-translational targeting system (Fröderberg et al., 2004). In the co-translational targeting system, the signal recognition particle (SRP) binds to the nascent peptide and leads the complex (nascent peptide + ribosome) to a membrane protein FtsY along with ribosomes; then, this SPR subsequently leads the nascent peptide to the channel complex (SecYEG). In the post-translational targeting system, the translation-finished peptide binds to SecB and SecA to reach the SecYEG channel (Singh et al., 2014). Once the linear peptide passes through the SecYEG channel, the signal peptide is cleaved by Type I signal peptidase and the protein is released from the membrane (Schallenberger et al., 2012).

The Tat-pathway is a twin-arginine translocation pathway with a conserved twin-arginine motif (RR) in the signal peptide (Figure 2C). The basic structure of the Tat system is divided into two complexes: a docking complex and a pore complex. The docking complex (TatB and TatC) recognizes the RR motif of the Tat signal peptide in the folded protein. Then, the folded protein is translocated across the active pore complex (TatA), with a structural change of the docking complex (Goosens et al., 2015).

### Examples of recombinant protein secretion in *C. glutamicum*

The production of a recombinant protein in *C. glutamicum* by a protein secretion system (the secretion of α-amylase from *Bacillus amyloliquefaciens* using the Sec system) was first reported by Smith et al. (Smith et al., 1986). Subsequently, protease, transglutaminase, green fluorescent protein (GFP), subtilisin, and endoglucanase have been produced in the *C. glutamicum* secretion system (Table 3).

**Table 3:** Examples of protein secretions in *Corynebacterium glutamicum*.

**Proteins**	Secretion system/resource^*^	**Resource**	**Producer**	**Secreted protein titer (g/L_medium_)**	**References**
**SEC SYSTEMS**					
Subtilisin (AprE)	Native	*Bacillus subtilis*	AS019 *+aprE*	0.0005	Billman-Jacobe et al., 1995
Protease (BprV)	AprE/B.s.	*Dichelobacter nodosus*	AS019 + BprV	0.0025	Billman-Jacobe et al., 1995
Protease (SAM-P45)	CspA/C.a	*Streptomyces albogriseolus*	ATCC13869 + SAM-P45	78 U/L	Kikuchi et al., 2003
Transglutaminase (MTG)	CspA/C.a	*Streptomyces mobaraense*	ATCC13869 + MTG	0.235	Kikuchi et al., 2003
Human epidermal growth factor (hEGF)	CspA/C.a	Human	YDK010 + hEGF	0.156	Date et al., 2006
Endoxylanase (XynA)	Porin B (PorB)/C.g	*Streptomyces coelicolor*	ATCC 13032 + XynA	0.615	An et al., 2013
Singlechain variable fragment (scFv)	Porin B (PorB)/C.g	*Escherichia coli*	ATCC 13032 + M18 scFv (codon-optimized)	0.068	Yim et al., 2014
Fab fragment of Human anti-HER2	CspA/C.a	Human	ATCC 13032 + Fab (H+L)	0.057	Matsuda et al., 2014
Endoxylanase (XynA)	Cg1514/C.g	*Streptomyces*	ATCC 13032 + XynA	1.07	Yim et al., 2016
α-amylase (AmyA)	Cg1514/C.g	*Streptococcus bovis*	ATCC 13032 + AmyA	0.78	Yim et al., 2016
Camelid antibody fragment (VHH)	Cg1514/C.g	Camelid	ATCC 13032 + CAb	1.58	Yim et al., 2016
α-amylase (AmyE)	CgR_2070/C.g	*Bacillus subtilis*	14067 + AmyE	103.24 U/mg	Jia et al., 2018
**TAT SYSTEMS**					
Endoglucanase (Clocel3242)	TorA*/*E.c	*Clostridium cellulovorans*	ATCC 13032 + Clocel3242	0.178	Tsuchidate et al., 2011
GFP	CgR0949/C.g	*Aequorea coerulescens*	R + AcGFP1	0.058	Teramoto et al., 2011
Sorbitol-xylitoloxidase (SoXy)	TorA*/*E.c	*Streptomyces coelicolor*	ATCC13032 + SoXy	–	Scheele et al., 2013
α-amylase	TorA/C.g	*Bacillus licheniformis*	BL-1 + pBlAmyS	0.49	Lee et al., 2014

* *B.s, Bacillus subtilis; C.a, Corynebacterium ammoniagenes; C.g, Corynebacterium glutamicum; E.c, Escherichia coli*.

Some studies have shown that eukaryotic proteins (such as human or camelid proteins) and microbial proteins could be successfully expressed in *C. glutamicum*. Yim et al. produced 68 mg/L_medium_ of a single-chain variable fragment (scFv) with anthrax toxin as an antigen in a Sec system by codon optimization using a strong promoter (Yim et al., 2014). When the same M18 scFv was expressed in *E. coli*, a slightly higher level of the protein was obtained (89.8 mg/L_medium_). Nevertheless, the use of *C. glutamicum* may be safer for drugs such as antibodies because endotoxins are not produced by a GRAS host, unlike the case of *E. coli*, and the secreted proteins are stable because there is no extracellular protease activity. Gram-positive bacteria such as *C. glutamicum* have no outer membrane; thus, target proteins need to pass through only one membrane to move out of the cell (van Wely et al., 2001). In addition, yeast cells that can glycosylate proteins when producing full-length antibodies are mainly used. However, in contrast to the glycosylation system of mammalian cells, yeast cells have a mannose-rich glycosylation system; thus, they are often not suitable for use in medicine. In particular, post-translational modifications such as glycosylation in *Pichia pastoris* often lead to unexpected protein structure and function (Dai et al., 2015). Nevertheless, *C. glutamicum* may be advantageous as a host for the expression of antibody fragments such as the scFv and Fab (antigen-binding fragment), which do not require glycosylation (Yim et al., 2014). Matsuda et al. produced 57 mg/L_medium_ of an Fab fragment of anti-human epidermal growth factor receptor 2 (anti-HER2) using the Sec-secretion system with a cell wall protein-deficient *C. glutamicum* strain (Matsuda et al., 2014). This was based on the formation of an intermolecular disulfide bond when the heavy and light subunits of anti-HER2 Fab fragments were present at the same time. In another study, Date et al. reported the production of 156 mg/L_medium_ of an active human epidermal growth factor (hEGF) with six cysteine residues that form three disulfide bonds, using the Sec-secretion system in *C. glutamicum* (Date et al., 2006). Therefore, *C. glutamicum* is an attractive secretory expression host for the production of medicinal proteins containing disulfide bonds as well as heterologous enzymes.

Efforts have also been made to introduce new signal peptides in *C. glutamicum*. An analysis of the secretion of *C. glutamicum* at high cell densities showed that the most abundant protein (51% of extracellular proteins) in the culture supernatant was a hypothetical protein encoded by *cg1514*. Using the promoter and signal peptide of the Cg1514 protein, three target proteins [endo-colanicol A, 1.07 g/L_medium_; *S. bovis* α-amylase, 0.78 g/L_medium_; camelid antibody fragment (VHH) for human lysozyme, 1.58 g/L_medium_] were produced (Yim et al., 2016). These results suggest that Cg1514-derived expression and secretion signals may be particularly effective in the production of secretory proteins from *C. glutamicum*.

Although not as common as the Sec system, there have been attempts to secrete proteins such as GFP or α-amylase using the Tat system in *C. glutamicum*. In particular, the Tat system is sometimes necessary because protein folding and the insertion of some cofactors into the proteins must occur in the cytoplasm. As the Tat system can transduce the substrates in a fully collapsed state through the cytoplasmic membrane, the use of the Tat-pathway for enzyme secretion has been investigated. To this end, FAD cofactor-containing sorbitol-xylitol oxidase (SoXy), which is a cytosolic enzyme of *Streptomyces coelicolor*, was expressed in *C. glutamicum* using the Tat secretion system (Scheele et al., 2013). This study demonstrated that heterologous proteins containing cofactors can also be produced using the *C. glutamicum* secretion system.

## Genetic tools for protein expression in *C. glutamicum*

To express recombinant proteins efficiently for the amino acid, food, and pharmaceutical industries, it is necessary to precisely control the expression of genes and to optimally control the metabolic flow toward the target protein or amino acid. Therefore, to produce the target protein efficiently, it is important to do the following: (1) optimize the promoter to increase expression efficiency, (2) construct a plasmid vector for various kinds of proteins, (3) construct an efficient protein-secretion pathway, and (4) design a *C. glutamicum* bioreactor culture system for high-yield production.

There have been attempts to increase the yield of expression systems, by using promoters (Table 4), which are mainly used for the production of amino acids and industrial enzymes with *C. glutamicum* as a host. The selection of optimal promoter and regulatory sequences is essential for producing useful products in living organisms. Promoters that are mainly used in *C. glutamicum* include several inducible promoters such as P_*lacUV*5_, P_*tac*_, P_*trp*_, P_*araBAD*_, P_*trc*_,, and the phage P_R_/P_L_ promoter from *E. coli* (Rytter et al., 2014). However, due to the low isopropyl-β-D-thiogalactopyranoside (IPTG) permeability of *C. glutamicum*, an IPTG-inducible expression system would have a lower expression level in *C. glutamicum* than in *E. coli*. Therefore, studies have been carried out to improve the promoter core sequence and membrane permeability of *C. glutamicum* and to increase gene expression by the site-directed mutagenesis. For example, a single-site mutation of the wild-type lac promoter has been used to enhance its protein expression level (Brabetz et al., 1991). In addition, the tac-M primer for constructing the tac promoter was found to increase the promoter activity following a mutation at the −10 region (Xu et al., 2010).

**Table 4:** Examples of inducible and constitutive promoters in *Corynebacterium glutamicum*.

**Promoter**	**Description**	**References**
**INDUCIBLE PROMOTERS**		
P_lac*UV*5_	IPTG inducible promoter	Brabetz et al., 1991
P*_*tac*_*	IPTG inducible promoter	Billman-Jacobe et al., 1994
P*_*trc*_*	IPTG inducible promoter	Kirchner and Tauch, 2003
P*_*prpB*_*	Propionate inducible promoter	Lee and Keasling, 2006
P_aceA/**aceB**_	Acetate-inducible promoter	Cramer et al., 2006
P_gntP/**gntK**_	Gluconate inducible promoter	Letek et al., 2006
P_CJ1OX2_	42°C inducible promoter	Park et al., 2008
P_tac−*M*_	Derived from the tac promoter, IPTG inducible promoter	Xu et al., 2010
P_malE1_, P*_*git*1_*	Maltose, Gluconate inducible promoter	Okibe et al., 2010
P_BAD_	Arabinose inducible promoter	Zhang et al., 2012
SPLs	Synthetic promoter libraries, IPTG-inducible	Rytter et al., 2014
P_4−N14_	Engineering the endogenous SigB-dependent promoter toward enhanced activity, stationary-phase gene expression system	Kim et al., 2016
**CONSTITUTIVE PROMOTERS**		
P*_*cspB*_*	Promoter of *cspB* gene, encoding glyceraldehyde-3-phosphate dehydrogenase	Peyret et al., 1993
P*_*aprE*_*	Promoter of *Bacillus subtilis* subtilisin (*aprE*)	Billman-Jacobe et al., 1995
P_180_	Isolated promoter from *Corynebacterium glutamicum* genome library	Park et al., 2004
P*_*sod*_*	Promoter of *sod* gene, encoding superoxide dismutase	Becker et al., 2005
P*_*dapA*_*	Promoter of *dapA* gene, not known to be prone to transcriptional control	van Ooyen et al., 2012
P*_*porB*_*	Promoter of *porB* gene, encoding porin B in *Corynebacterium glutamicum*	An et al., 2013
P*_*ilvC*_*	Promoter of *ilvC* gene, encoding ketol-acid reductoisomerase	Kang et al., 2014
P_L10_, P_L26_, P_I16_, P_I51_, P_H30_, P_H36_	Fully synthetic promoter library consisting of 70-bp random sequences in *Corynebacterium glutamicum*	Yim et al., 2013; Oh et al., 2015

An auto-inducible promoter is a promoter that expresses proteins according to variables such as the nutrient type/concentration, oxygen level, pH level, and cell growth stage (Chou et al., 1995); this is advantageous for producing recombinant proteins on an industrial scale. Kim et al. (2016) engineered the SigB-dependent cg3141 promoter in *C. glutamicum* to develop an auto-inducible promoter system that is capable of expressing recombinant proteins during the transition phase between the log phase and the stationary phase of the cells. As a result, the model protein, glutathione S-transferase, was successfully produced on a lab-scale bioreactor (5 L) by introducing the P_4−N14_ promoter (Kim et al., 2016).

The use of constitutive promoters is advantageous because they do not require expensive reagents for induction or optimized induction conditions (Yim et al., 2013). Constitutive promoters derived from the genome of *C. glutamicum*, such as P_*sodA*_, P_*gapA*_, P_*eftu*_, and P_*cspB*_, are known to have high expression levels. However, the strength of the promoters cannot be directly compared and the use of strong promoters can also be affected by other genetic elements such as the 5′-untranslated region (5′-UTR) (Teramoto et al., 2011) and transcription initiation region (TIR) (Yim et al., 2013). Therefore, the selection of an optimal promoter is required because a strong promoter alone does not guarantee high protein expression. Yim et al. have developed the first synthetic promoter in *C. glutamicum* (Yim et al., 2013). Sequences including P_L10_, P_L26_, P_I16_, P_I51_, P_H30_, and P_H36_ were selected from the promoter library, which consisted of 70 randomly chosen nucleotide sequences. Among them, P_H36_ was the strongest promoter, and it successfully induced the expression of antibody fragments and endoxylanase (746 mg/L_medium_), as model proteins. Appropriate expression vectors and promoters are also important for increasing the yield of recombinant proteins. Currently, several *C. glutamicum*–*E. coli* shuttle expression vectors are being widely used (Table 5).

**Table 5:** Examples of expression vectors in *C. glutamicum*.

**Vector**	**Size (kb)**	**Replicon**	**Copy number per cell**	**Selection marker**	**Promoter, regulatory gene**	**Induction conditions (Conc.)**	**References**
pEKEx1	8.2	pBL1	10–30	Km^r^	P*_*tac*_, lacI^*q*^*	IPTG (0.2 mM)	Eikmanns et al., 1991
pXMJ19	6.6	pBL1	10–30	Cm^r^	P*_*tac*_, lacI^*q*^*	IPTG (1 mM)	Anglana and Bacchetti, 1999
pBKGEXm2	7.3	pBL1	10–30	Km^r^	P*_*tac*_, lacI^*q*^*	IPTG (1 mM)	Srivastava and Deb, 2002
pCRA1	5.3	pBL1	10–30	Cm^r^	P*_*lac*_*	Constitutive	Nakata et al., 2003
pCRA429	4.3	pBL1	10–30	Cm^r^	P*_*tac*_*	Constitutive	Suzuki et al., 2009
pDXW-8	9.6	pBL1	10–30	Km^r^	P*_*tac*_, lacI*^PF104^	IPTG (1 mM)	Xu et al., 2010
pEC901	8.5	pCG1	30	Km^r^	P_L_/P_R_ (λ), cI857	40°C	Makoto Tsuchiya, 1988
pZ8-1	7.0	pCG1	30	Km^r^	P*_*tac*_*	Constitutive	Dusch et al., 1999
pVWEx1	8.5	pCG1	30	Km^r^	P*_*tac*_, lacI^*q*^*	IPTG (1 mM)	Peters-Wendisch et al., 2001
pSL360	6.5	pCG1	30	Km^r^	P_180_	Constitutive	Park et al., 2004
pECXK99E	7.0	pGA1	30	Km^r^	P*_*tac*_, lacI^*q*^*	IPTG (0.5 mM)	Kirchner and Tauch, 2003
pTRCmob	6.4	pGA1	30	Km^r^	P*_*trc*_*	IPTG (0.2 g/L_medium_)	Liu et al., 2007
pAPE12	4.6	pNG2	<10	Km^r^	P*_*tac*_, lacI^*q*^*	IPTG (0.15 g/L_medium_)	Guillouet et al., 1999

## Conclusion

*C. glutamicum* can be used as an industrial L-glutamate and L-lysine producer. In addition, various types of recombinant proteins can be expressed in *C. glutamicum*, which has been used for several decades for the production of microbial enzyme. Furthermore, *C. glutamicum* has been used to increase yields, develop new anchoring systems, and signal peptides (for the efficient production of biochemicals and nutraceuticals, enzymes, medicinal proteins, and biopolymers), and screen synthetic promoters of various strengths. However, using *C. glutamicum* as an expression host has several disadvantages when compared with using *E. coli* as an expression host: (1) a much lower transformation efficiency, (2) fewer available expression systems, and (3) lower yields for some proteins, especially antibodies. Therefore, further studies are necessary to develop various tools to enhance protein yields and reduce manufacturing costs. Recent advances in bioinformatics, such as next-generation sequencing (NGS), RNA-seq, and proteomics, would provide more information on the protein production pathways in *C. glutamicum*.

## Author contributions

ML wrote the manuscript and PK supervised. All authors have made intellectual contributions to the work, and approved it for publication.

### Conflict of interest statement

The authors declare that the research was conducted in the absence of any commercial or financial relationships that could be construed as a potential conflict of interest.
